# Comparative analysis of endovascular treatment methods for anterior choroidal aneurysms: single center study with 80 aneurysms

**DOI:** 10.1007/s00701-025-06647-9

**Published:** 2025-09-01

**Authors:** Yigit Can Senol, Halis Emre Ciftci, Zeynep Gence Oz, Dilara Duman, Bige Sayin, Ilkay Akmangit, Musa Onur Ozbakir, Denizhan Divanlioglu, Ahmet Deniz Belen, Ergun Daglioglu

**Affiliations:** 1 Department of Neurosurgery, University of California San Francisco, San Francisco, CA USA; 2 Department of Neurosurgery, University of Health Sciences Ankara Bilkent City Hospital, Ankara, Turkey; 3 Department of Radiology, University of Health Sciences Ankara Bilkent City Hospital, Ankara, Turkey

**Keywords:** Anterior choroidal artery, Aneurysm, Endovascular

## Abstract

**Purpose:**

Anterior choroidal artery (AChoA) aneurysms are rare and pose a significant treatment challenge due to the artery’s small caliber and critical vascular territory. Endovascular treatment (EVT) has become a preferred approach, but optimal management strategies remain debated. This study compares the efficacy and safety of different EVT techniques, including primary coiling, stent-assisted coiling (SAC), and flow diversion (FD), in treating AChoA aneurysms.

**Methods:**

Patients were categorized by endovascular technique, aneurysm morphology, and rupture status. Angiographic occlusion rates were assessed using the Raymond-Roy Occlusion Scale (RROS), and clinical outcomes were measured via the Modified Rankin Scale (mRS) at discharge and follow-ups. Statistical analyses compared occlusion rates, procedural complications, and functional outcomes among treatment groups.

**Results:**

In this study, 60 patients with 80 anterior choroidal artery aneurysms were treated. Among these, 44 aneurysms (55%) were classified as dependent, meaning the choroidal branch arose from the aneurysm dome or neck, while 36 aneurysms (45%) were independent, originating from the carotid artery near the choroidal branch. Primary coiling was used in 29 cases, stent-assisted coiling (SAC) in 21, and flow diversion (FD), with or without additional coiling, in 30 cases. Complete occlusion rates were significantly higher with SAC (83.3%) and FD (76.1%) compared to primary coiling (31.8%) (p < 0.05). Flow diversion was associated with more technical complications (25%), and ischemic events were more common in dependent aneurysms (p < 0.05). Importantly, no cases of symptomatic AChoA occlusion occurred after FD treatment. The overall mortality rate was 5%, with all deaths occurring in the primary coiling group among patients with ruptured aneurysms.

**Conclusion:**

EVT of AChoA aneurysms is effective, with SAC and FD demonstrating superior occlusion rates compared to primary coiling. FD carries a higher risk of technical complications but maintains AChoA patency. To optimize outcomes, treatment choice should be guided by aneurysm morphology and patient risk factors.

## Introduction

Anterior choroidal artery (AChoA) aneurysms are uncommon, accounting for just 2–5% of all intracranial aneurysms [1, 12]. Despite their low incidence, their treatment remains highly challenging due to the delicate nature of the AChoA and its critical vascular supply to eloquent brain structures [16, 18]. Even minor disruptions to this artery can lead to severe neurological deficits, complicating treatment decisions by necessitating a balance between aneurysm occlusion and the preservation of blood flow of AChoA [5, 20].

Endovascular techniques have gained popularity as minimally invasive alternatives but ensuring complete aneurysm occlusion while preserving AChoA patency remains a challenge. The management of AChoA aneurysms is particularly difficult because both surgical and endovascular approaches carry inherent risks. While surgical clipping provides direct visualization and the potential for aneurysm occlusion, it is often associated with a higher risk of ischemic complications due to the artery’s small caliber and deep location within the brain [8, 15, 16].

To address these challenges, several endovascular treatment modalities have been used to enhance both safety and efficacy. Primary coiling [7, 9], flow diverters (FD) [6, 11], stent-assisted coiling (SAC) [4, 14], and combined FD with coiling [3, 13] each offer distinct advantages depending on aneurysm morphology and rupture status. However, the choice of treatment is often individualized based on clinical and anatomical factors, aiming to balance safety and aneurysm occlusion. This study aims to compare the clinical and angiographic outcomes of different EVT strategies in the treatment of AChoA aneurysms.

## Methods

This retrospective study was conducted in accordance with the ethical standards of the institutional and national research committee, as well as the 1964 Helsinki Declaration and its later amendments or comparable ethical standards. The clinical research ethics board of Ankara Bilkent City Hospital approved this study (decision number E1-20–1444).

### Patient population

This retrospective study utilized data extracted from a prospectively maintained institutional aneurysm treatment database. Patients were included if they had at least one AChoA aneurysm treated endovascularly at our institution between March 2011 and August 2023. In cases with multiple aneurysms, patients were included only if the AChoA aneurysm was treated in isolation or could be clearly identified among multiple aneurysms. Exclusion criteria included surgically treated AChoA aneurysms, conservative cases(observed without treatment), and incomplete clinical or radiological data. Comorbidities were not considered exclusionary but are acknowledged in the limitations section as potential confounding factors. For each patient, we recorded demographic data, clinical presentation, location of the aneurysm, endovascular treatment method, perioperative and postoperative complications, early clinical results, and long-term clinical and angiographic outcomes. Two authors retrospectively collected the data (Y.C.S. and H.E.C).

### Classification of AChoA aneurysms

The analysis of the aneurysm characteristics was performed by the senior neurointerventionalist (B.S.) and a senior neurosurgeon (M.O.O.). Aneurysms originating from the carotid artery and very close to the choroidal artery were defined as independent aneurysms(independent from the choroidal branch) and where choroidal artery segment arises from aneurysm neck or dome as dependent aneurysms as reported in the previous study [2].

### Endovascular technique

All endovascular procedures were performed under general anesthesia. Prior to the procedure, detailed evaluations of three-dimensional DSA images were conducted. During the acute SAH phase, all patients initially underwent primary coiling; if there was a coil migration, stent assistance was used. In this particular patient group, our group did not use balloon assistance in the patient cohort.

The choice of endovascular treatment strategy was individualized based on several factors, including aneurysm rupture status, morphology (e.g., neck width, dome size, dome-to-neck ratio), clinical condition of the patient, and device availability at the time of the procedure. In cases of ruptured aneurysms, primary coiling was the initial treatment of choice. However, if coil stability could not be achieved due to wide-neck morphology or coil migration, stent-assisted coiling (SAC) or flow diverter procedure was performed. Flow diversion was primarily reserved for unruptured, wide-neck aneurysms or used as a second-stage treatment in patients who initially underwent coiling during the acute SAH phase.

A triaxial system (Heety 8 F Long-sheath, Fargo mini [*Balt Extrusion, Montmorency, France*]) with a distal access catheter and Excelsior SL-10 (*Stryker, Kalamazoo, Michigan, USA*) was utilized for primary coiling, covering at least 70% of the AChoA aneurysm in the acute phase of SAH. If the aneurysm is not ruptured, the coiling density was aimed at 90% at least. In case of subarachnoid hemorrhage, partial coiling with or without stent assistance is preferred.

For the Flow diversion treatment, Silk Vista(*Balt,Montmorency,France*) and Surpass Evolve(*Stryker, Kalamazoo, Michigan*) flow diverters were used. Flow diverter choice is made by the availability of sizes. Before flow diversion, patients were medicated with dual anti-platelet(DAPT) drugs Clopidogrel(Loading Dose: 600 mg) or Prasugrel(Loading Dose: 30-60 mg) and Aspirin(Loading Dose 500 mg), maintained with tirofiban during the procedure. In cases with no premedication with DAPT before the procedure, a body weight-adapted bolus dose of intravenous tirofiban was given in addition to the loading doses mentioned above. Post-procedure, patients continued on dual antiplatelet therapy(Clopidogrel(1 × 75 mg) or Prasugrel(1 × 10 mg) and Aspirin(1 × 100 mg)) for one year, tailored to individual patient and aneurysm characteristics.

### Angiographic and clinical outcomes

Angiographic outcomes for endovascular treatment were evaluated using the Raymond Roy Occlusion scale(RROS) [10]. The assessment of aneurysm occlusion status was conducted by a senior neurosurgeon (E.D.) and a senior neuroradiologist (B.S.) based on digital subtraction angiography (DSA) findings immediately after the procedure. Follow-up angiographic evaluations were performed between 6 months to 1-year post-treatment to monitor aneurysm occlusion progression. Procedure-related complications were classified as perioperative aneurysm rupture, vascular access site complications, transient or permanent ischemic complications, hemorrhagic complications(a new hemorrhage in 72 h after the procedure), and technical complications such as iatrogenic vessel injury, stent/flow diverter shortening. Clinical outcomes were retrospectively assessed using the Modified Rankin Scale (mRS) at baseline, at discharge, and during the most recent follow-up visit to evaluate patient disability and functional recovery. These evaluations ensured a standardized approach to outcome assessment in endovascularly treated anterior choroidal artery aneurysms.

### Statistical analysis

Descriptive statistics were used to summarize continuous variables as mean ± standard deviation and categorical variables as frequencies and percentages. The Chi-square (χ^2^) test or Fisher’s exact test was applied to compare categorical variables. For continuous variables, Welch’s t-test was used for normally distributed data, while Wilcoxon rank-sum test was applied for non-parametric data.

Comparisons of angiographic outcomes (e.g., Raymond-Roy occlusion classification), clinical outcomes (e.g., mRS at discharge and follow-up), and complication rates (e.g., ischemic, hemorrhagic, technical) across treatment groups were performed using these categorical tests. Aneurysm morphology (independent vs. dependent), categorical data were similarly compared using the Chi-square or Fisher’s exact test based on distribution characteristics. Due to the relatively small sample size and notable baseline imbalances in rupture status and pre-treatment functional status, multivariate or adjusted analyses were not performed. The statistical analysis in this study was performed using SPSS version 28 (*IBM Corp., Armonk, NY, USA*).

## Results

### Patient demographics and aneurysm morphology

**Fig. 1:**
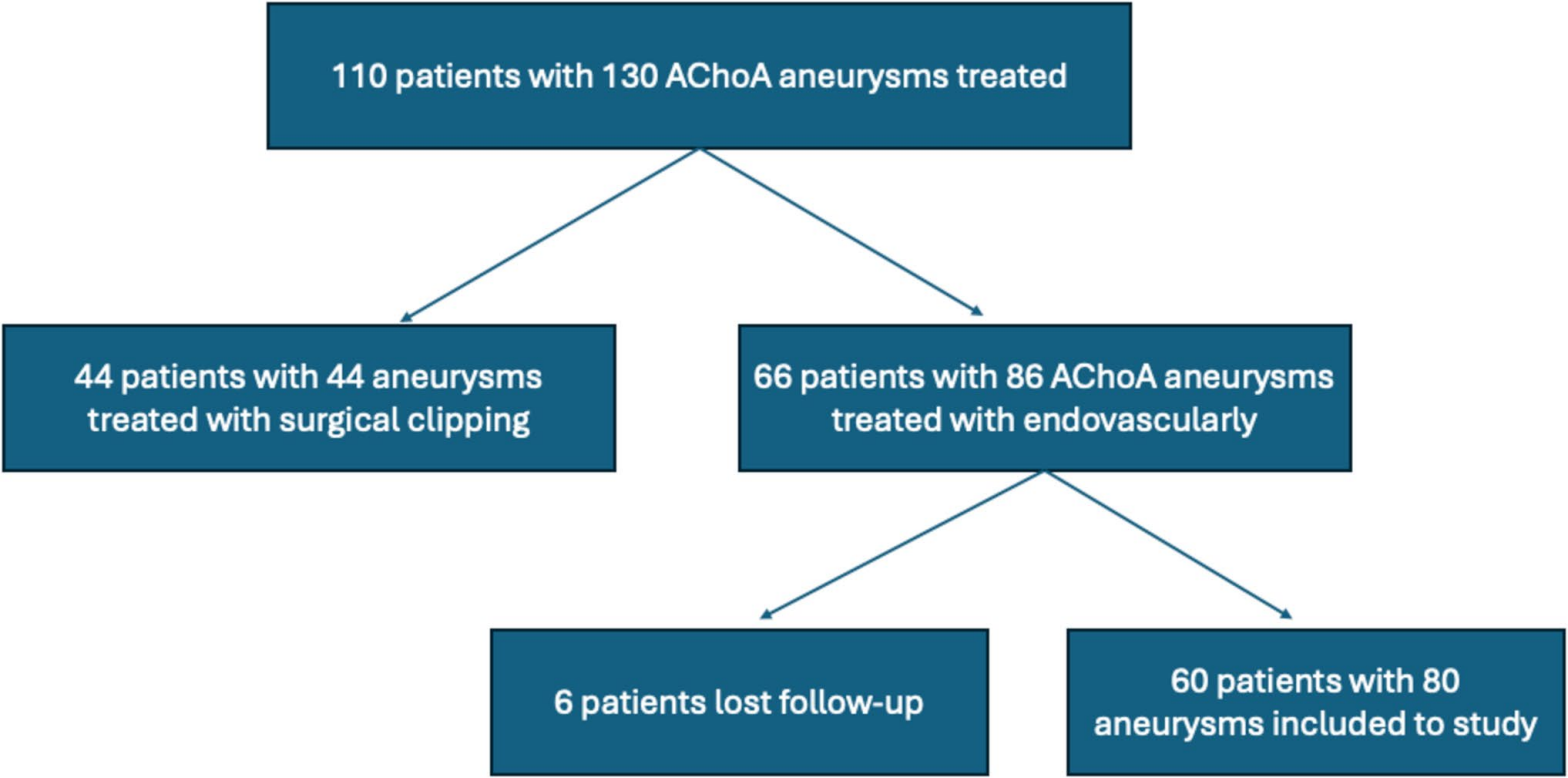
Flowchart of patient selection

A total of 110 patients with 130 anterior choroidal artery (AChoA) aneurysms were initially treated between 2011 and 2023. Of these, 44 aneurysms were treated with surgical clipping, and 86 aneurysms in 66 patients were treated endovascularly. Among the endovascular group, 6 patients were excluded due to loss of follow-up data. The nature of the missing data was lack of post-procedural follow-up imaging and clinical records, which precluded outcome assessment. Thus, the final analysis included 60 patients with 80 AChoA aneurysms treated by endovascular techniques(Fig. 1). Among them, 32 patients (53.3%) were female and 28 (46.6%) were male. The youngest patient was 18 years old and the oldest was 73 years old. The median age at treatment was 58.0 years (IQR: 49.0–63.0) for primary coiling, 56.0 years (IQR: 40.2–74.7) for stent-assisted coiling, 53.5 years (IQR: 46.7–63.2) for flow diverter with coiling, and 52.0 years (IQR: 39.0–62.0) for flow diverter alone. Regarding aneurysm presentation, 39 aneurysms (48.7%) were ruptured, leading to subarachnoid hemorrhage (SAH), while 41 aneurysms (51.2%) were unruptured or diagnosed incidentally.

Of the 80 anterior choroidal artery aneurysms included, 39 (48.7%) were ruptured, presenting with subarachnoid hemorrhage (SAH), and 41 (51.3%) were unruptured or incidentally discovered. Treatment choice was influenced by rupture status—ruptured aneurysms were predominantly managed with primary or staged FD + coiling due to the urgency and need for immediate aneurysm occlusion, whereas unruptured aneurysms were more frequently treated electively with flow diversion or stent-assisted coiling based on anatomical complexity and long-term occlusion goals. All preoperative parameters are summarized in Table 1.

**Table 1:** Summary of preoperative patient groups

	Primary Coiling(N = 29)	Stent-Assisted Coiling(6)	Flow Diverter + Coiling(n = 20)	Flow Diverter(n = 25)	p—value
Age	56.3	58.2	54.1	49.7	0.256
Gender(Female)	11(38.3%)	3(50%)	10(50%)	17(68.4%)	0.181
AchoA Aneurysm Location(Right)	23(79.1%)	2(33.3%)	11(45%)	5(25%)	0.857
Dome Width	4.07 ± 1.91	4.87 ± 2.56	3.59 ± 1.73	2.73 ± 1.11	0.19
Dome Height	5.14 ± 3.08	5.43 ± 3.12	4.72 ± 3.36	3.06 ± 1.71	0.221
Neck Width	2.60 ± 1.06	2.74 ± 0.85	2.44 ± 1.23	2.30 ± 1.23	0.103
Dome/Neck Ratio	1.67 ± 0.68	1.46 ± 0.33	1.63 ± 0.75	1.40 ± 0.59	0.149
Follow-up Months	8.29 ± 6.19	40.67 ± 40.25	10.90 ± 7.22	11.58 ± 12.05	0.265
DSA Follow-Up	22(75.9)	4(84.4)	15(75.0)	6(100)	0.499
mRS 0–2 (%)	17/29 (58.6%)	4/6 (66.7%)	16/20 (80.0%)	23/25 (92.0%)	**0.036**
mRS 3–5 (%)	12/29 (41.4%)	2/6 (33.3%)	4/20 (20.0%)	2/25 (8.0%)	**0.036**
SAH—Ruptured (%)	23/29 (79.3%)	2/6 (33.3%)	9/20 (45.0%)	5/25 (20.0%)	** < 0.001**
Choroidal Type—Dependent (%)	17/29 (58.6%)	3/6 (50.0%)	12/20 (60.0%)	12/25 (48.0%)	0.781

### Angiographic outcomes

**Fig. 2:**
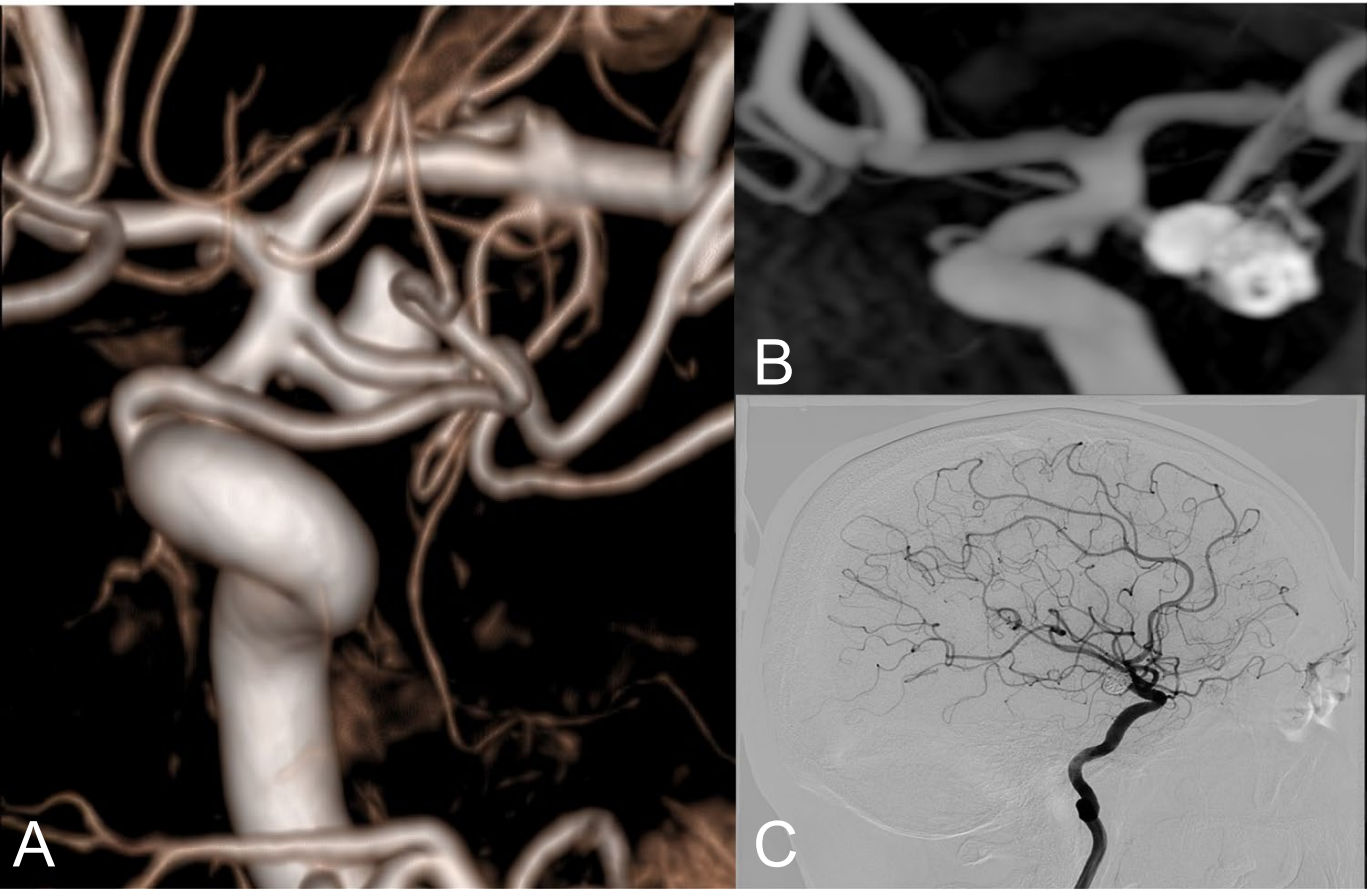
Panels (A–C) show aneurysms managed with primary coiling, where 3D angiography (**A**) and reconstructed postoperative angiography images (**B**) depict aneurysm morphology, and 6.^th^ month follow-up DSA (**C**) confirms coil placement with preserved anterior choroidal artery flow without residual filling

**Fig. 3:**
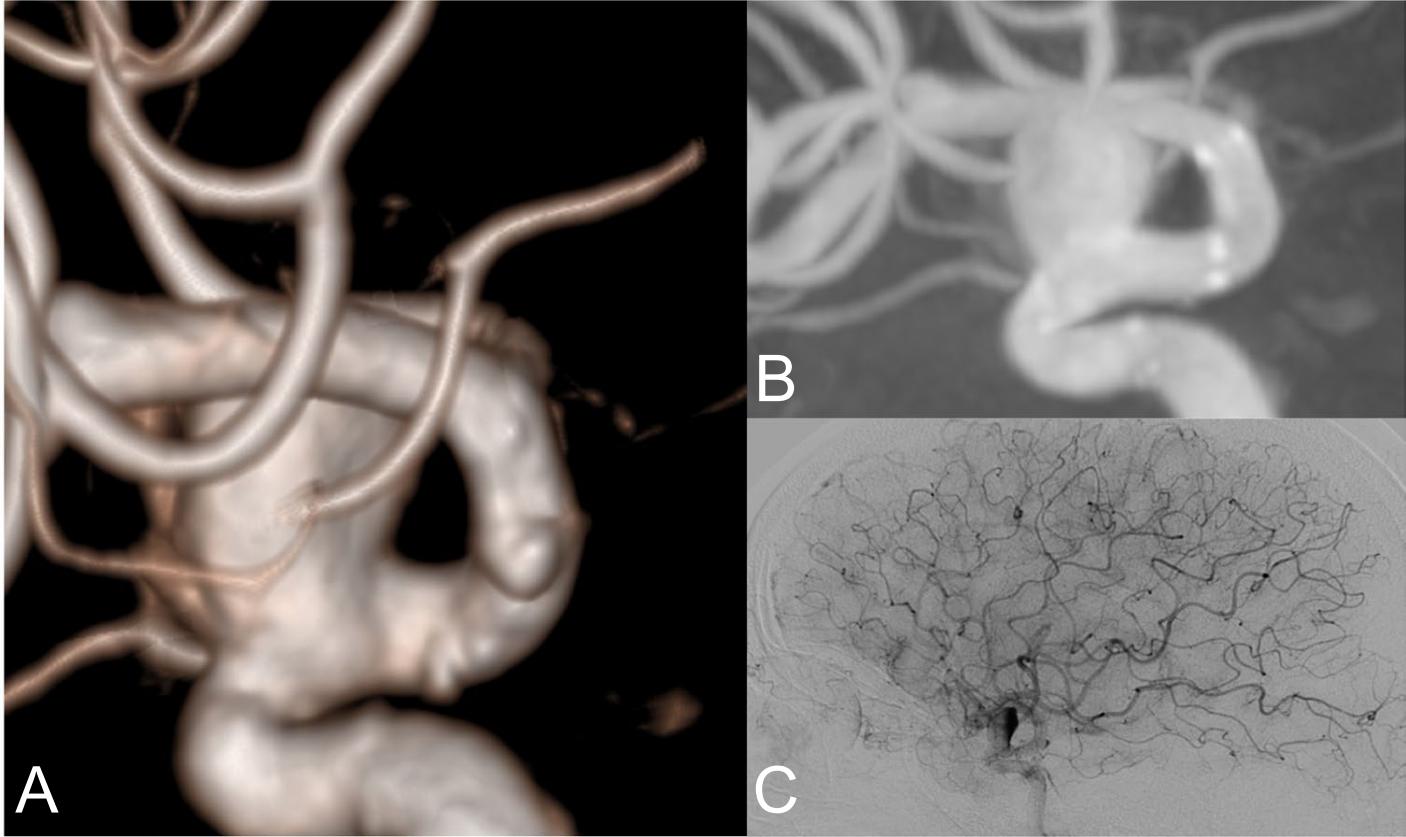
Panels (A–C) illustrate treatment with a flow diverter, with 3D reconstructed images (**A**) highlighting the dependent aneurysm neck with choroidal branch arising(arrow), angiographic reconstruction (**B**) showing flow diverter placement (arrows), and immediate DSA (**C**) demonstrating contrast hanging

**Fig. 4:**
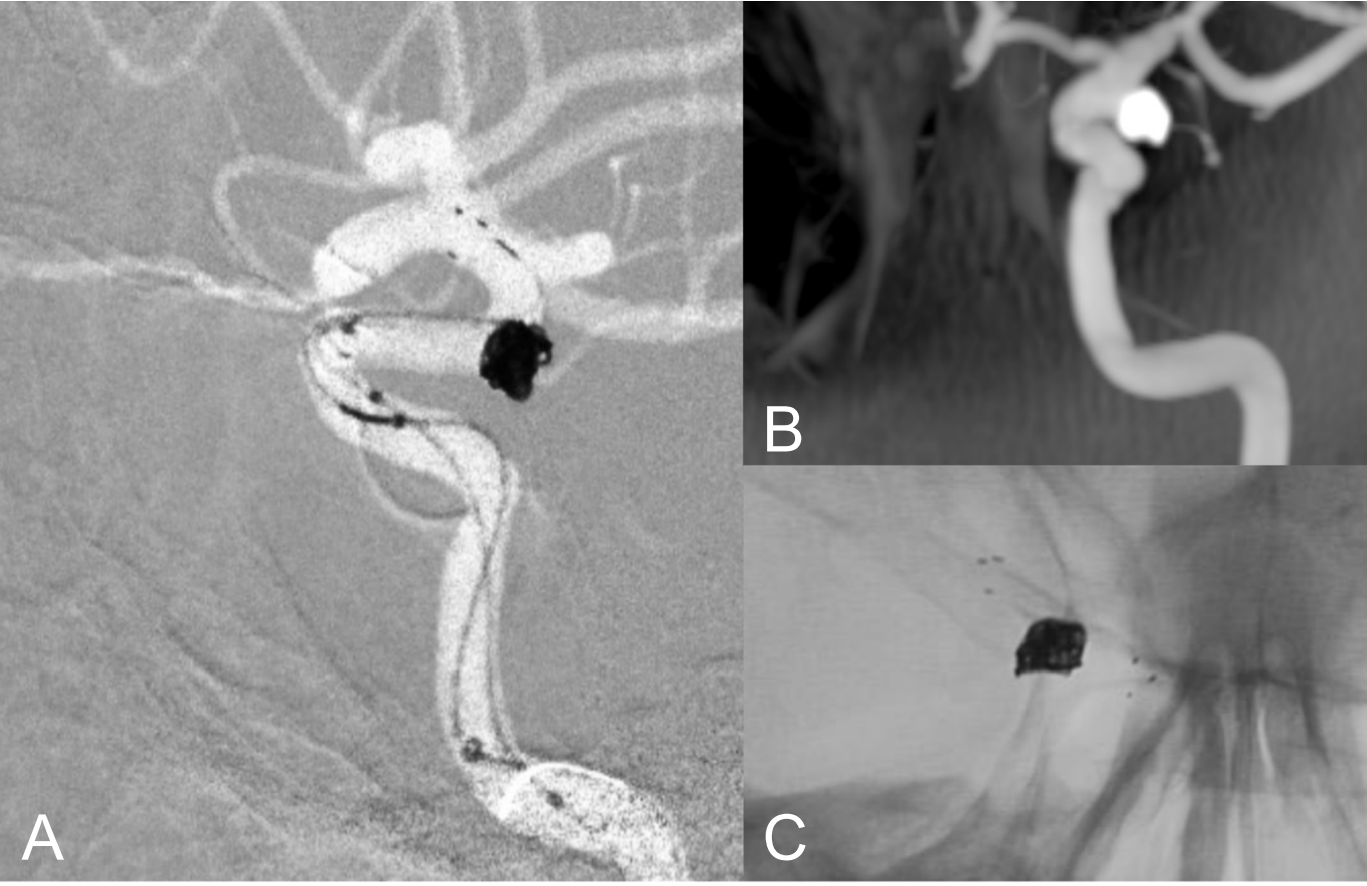
Panels (A–C) represent stent-assisted coiling, where subtracted road map (**A**) image demonstrates perioperative coil deployment, with preserving choroidal branch(arrow), native image (**B**) confirms coil stability, and 3DRA (**C**) shows complete occlusion with maintained arterial patency (arrow)

Follow-up angiographic data showed that complete occlusion was observed in a greater proportion of cases treated with flow diversion, with or without adjunctive coiling, compared to those treated with primary coiling (Fig. 2). Specifically, among flow-diverter-treated aneurysms (Fig. 3), 76.1% achieved complete occlusion, while the combination of flow diversion and adjunctive coiling resulted in 73.3% complete occlusion. Stent-assisted coiling (Fig. 4) also demonstrated high occlusion rates, with 83.3% of aneurysms achieving complete occlusion. Long-term follow-up showed a statistically significant difference in angiographic occlusion rates among treatment groups, with higher rates observed in patients treated with flow diversion techniques compared to those treated with primary coiling (*p* = 0.007). (Table 2).

**Table 2:** Clinical outcomes of endovascular treatment methods

	Primary Coiling	Stent-Assisted Coiling	Flow Diverter + Coiling	Flow Diverter	p—value
Transient Ischemic Attack (TIA)	3(10.3%)	1(16.7%)	4(20%)	5(20%)	0.751
Ischemic Complication	8(27.6)	1(16.7%)	1(5%)	5(4%)	**0.046**
Hemorrhagic Complication	2(6.9%)	0	0	0	0.307
Technical Complication	0	0	1(5%)	5(25%)	**0.034**
Retreatment	0	0	1(5%)	0	0.386
Long term Angiographic Occlusion (complete occlusion)	7 (31.8)	5 (83.3)	11 (73.3)	16 (76.2)	**0.007**
Post-tx discharge mRS 0–2	19 (65.5)	5 (83.3)	18 (90.0)	22 (88.0)	0.107
1 month mRS 0–2	22 (75.9)	5 (83.3)	20 (100)	22 (95.7)	**0.038**
3th-6th month mRS 0–2	11 (84.6)	6 (100)	17 (100)	19 (100)	0.082
1 st year mRS 0–2	4 (66.7)	4 (100)	9 (100)	10 (100)	**0.041**

### Clinical outcomes and complications

Functional outcomes were evaluated using the Modified Rankin Scale (mRS) at discharge, 1-month, 6-month, and 1-year follow-ups, with no statistically significant difference observed among treatment groups (*p-value: 0.046*). However, the primary coiling group showed a higher mortality rate (10.3%), primarily due to poor preoperative clinical status (mRS > 4).

Ischemic complications were significantly more frequent in dependent-type aneurysms compared to independent aneurysms (p < 0.05)(Table 3). Flow diversion was associated with a higher rate of technical complications, including device malposition or shortening, requiring additional stent or flow diverter placement in 25% of cases. Stent-assisted coiling and flow diversion techniques had the lowest incidence of periprocedural rupture and ischemic events, and no cases of complete anterior choroidal artery occlusion were reported following flow-diversion treatment. The overall mortality rate was 5% (3/60 patients), all within the primary coiling group due to SAH-related complications.

**Table 3:** Comparison of Complications in different AchoA Aneurysm types

	Independent (n = 44)	Dependent Type (n = 36)	p—value
Transient Ischemic Attack (TIA)	5(11.4%)	8(22.2%)	0.190
Ischemic Complication	2(4.5%)	9(25.0%)	**0.008**
Hemorrhagic Complication	2(5.0%)	0(0%)	0.195
Technical Complication	3(6.8%)	3(8.3%)	0.798
Retreatment	1(2.3%)	0(0%)	0.363

## Discussion

Our study evaluated the outcomes of various EVT techniques for AChoA aneurysms, with a focus on angiographic occlusion rates, clinical outcomes, and procedural complications. We found that flow diversion techniques were associated with higher aneurysm occlusion rates compared to primary coiling in this cohort. However, flow diverters were associated with a higher rate of technical complications. Importantly, no cases of complete AChoA occlusion were observed after flow-diversion treatment—an essential finding given the high risk of ischemic injury in this territory. While the current literature generally discourages the use of flow diverters in ruptured AChoA aneurysms due to limited data, our study suggests that primary coiling, stent-assisted coiling (SAC), and flow diversion with adjunctive coiling may all be viable treatment options in selected cases of ruptured AChoA aneurysms.

Beyond angiographic outcomes, clinical results and complication profiles offer important context for treatment selection. Although there were no statistically significant differences in functional outcomes (mRS scores) among treatment groups, mortality was highest in the primary coiling group (10.3%), primarily due to poor preoperative neurological status in patients presenting with SAH. Ischemic complications were significantly more common in dependent aneurysms than in independent aneurysms (25% vs 4.5%, p < 0.05), underscoring the relevance of aneurysm morphology in EVT planning. Flow diversion had the highest rate of technical complications, with 25% of cases requiring additional intervention due to device malposition or shortening. Nevertheless, both stent-assisted coiling and flow diversion demonstrated the lowest rates of periprocedural rupture and ischemic events, consistent with prior reports in the literature. [13, 17]

Prior literature has reported that primary coiling alone may not provide durable aneurysm occlusion, particularly in wide-neck aneurysms [19, 21]. Our findings reinforce this, as flow diversion and adjunctive coiling yielded significantly higher long-term occlusion rates than primary coiling (%73.3 vs 31.8%, p = 0.023), aligning with multi-institutional studies showing that flow-diverting stents achieve substantial aneurysm occlusion while preserving branch patency [17]. Concerns regarding the risk of AChoA occlusion with flow-diverter placement have been reported [6], yet we observed no symptomatic infarctions or vessel occlusions post-procedure, supporting findings that AChoA patency is typically maintained due to collateral circulation and hemodynamic adaptation. The increased ischemic risk associated with dependent aneurysms further highlights the need for individualized treatment selection based on aneurysm morphology and patient-specific risk factors.

Given the complexity of AChoA aneurysms, EVT strategies should aim to minimize ischemic complications while maximizing occlusion durability. Flow diversion, alone or with adjunctive coiling, appears to provide the best balance of efficacy and safety in appropriately selected cases, though careful device deployment and follow-up imaging are essential due to the increased risk of technical complications. Stent-assisted coiling remains a viable option for wide-neck aneurysms where flow diversion is unsuitable, offering favorable occlusion rates with a lower procedural risk. Primary coiling is associated with a higher likelihood of rebleeding and retreatment rates, particularly in ruptured aneurysms. Treatment decisions should, therefore, be guided by aneurysm characteristics, patient neurological status, and operator expertise, with a preference for staged approaches when necessary.

This study has several limitations. First, its retrospective and single-center design may limit the generalizability of the findings, as treatment decisions and procedural techniques could vary across institutions. Second, the relatively small sample size, despite being one of the larger series on AChoA aneurysms, reflects the rarity of these lesions and may impact statistical power, particularly in subgroup analyses. Third, the lack of long-term follow-up beyond one year for all patients may underestimate late aneurysm recurrence, delayed ischemic events, or flow-diverter-related complications. Fourth, treatment selection was not randomized, which could introduce selection bias, as patients with different aneurysm morphologies, rupture statuses, and baseline functional scores (mRS) were managed with different EVT strategies. These baseline imbalances may limit the validity of direct comparisons between treatment groups, and findings should be interpreted as observational and hypothesis-generating rather than definitive. While the study includes comparative elements, it is best framed as a descriptive observational analysis reflecting real-world treatment patterns rather than a comparative efficacy study. Sixth, occlusion status was evaluated independently by two neurointerventionalists; however, inter-rater reliability was not formally assessed, which may introduce a degree of subjectivity in outcome classification. Results are interpreted as observational and hypothesis-generating rather than confirmatory. A two-tailed p-value < 0.05 was considered statistically significant. Finally, the study does not include a direct comparison with microsurgical treatment, which remains an alternative for select cases, particularly those with complex aneurysm morphology. Future prospective, multicenter studies with longer follow-up periods are needed to validate these findings and optimize EVT decision-making for AChoA aneurysms.

## Conclusion

Endovascular treatment of anterior choroidal artery aneurysms, as observed in this retrospective cohort of 60 patients treated at a high-volume center, was associated with acceptable safety and occlusion rates, though these findings should be interpreted with caution due to the study’s non-randomized design and limited generalizability. While technical complications were more familiar with flow diverters, they did not result in symptomatic AChoA occlusion, supporting their use as a viable treatment option. Dependent-type choroidal aneurysms were associated with higher ischemic complication rates, underscoring the importance of aneurysm morphology in EVT planning. Future research should focus on optimizing treatment selection criteria and evaluating long-term outcomes to further refine management strategies for these challenging lesions.

## Data Availability

No datasets were generated or analysed during the current study.
